# Synchronized Optical and Acoustic Droplet Vaporization for Effective Sonoporation

**DOI:** 10.3390/pharmaceutics11060279

**Published:** 2019-06-14

**Authors:** Wei-Wen Liu, Sy-Han Huang, Pai-Chi Li

**Affiliations:** 1 Graduate Institute of Biomedical Electronics and Bioinformatics, National Taiwan University, Taipei 106, Taiwan; wwliu0801@gmail.com (W.-W.L.); seanhuang26@gmail.com (S.-H.H.); 2 Department of Electrical Engineering, National Taiwan University, Taipei 106, Taiwan

**Keywords:** nanodroplets, cavitation, acoustic droplet vaporization, optical droplet vaporization, sonoporation

## Abstract

Inertial cavitation-based sonoporation has been utilized to enhance treatment delivery efficacy. In our previous study, we demonstrated that tumor therapeutic efficacy can be enhanced through vaporization-assisted sonoporation with gold nanodroplets (AuNDs). Specifically, the AuNDs were vaporized both acoustically (i.e., acoustic droplet vaporization, ADV) and optically (i.e., optical droplet vaporization, ODV). A continuous wave (CW) laser was used for ODV in combination with an ultrasound pulse for ADV. Although effective for vaporization, the use of a CW laser is not energy efficient and may create unwanted heating and concomitant tissue damage. In this study, we propose the use of a pulsed wave (PW) laser to replace the CW laser. In addition, the PW laser was applied at the rarefaction phase of the ultrasound pulse so that the synergistic effects of ADV and ODV can be expected. Therefore, a significantly lower laser average power can be expected to achieve the vaporization threshold. Compared to the CW laser power at 2 W/cm^2^ from the previous approach, the PW laser power was reduced to only 0.2404 W/cm^2^. Furthermore, we also demonstrate in vitro that the sonoporation rate was increased when the PW laser was applied at the rarefaction phase. Specifically, the vaporization signal, the inertial cavitation signal, and the sonoporation rate all displayed a 1-µs period, which corresponded to the period of the 1-MHz acoustic wave used for ADV, as a function of the relative laser delay. The increased sonoporation rate indicates that this technique has the potential to enhance sonoporation-directed drug delivery and tumor therapy with a lower laser power while keeping the cell death rate at the minimum. Photoacoustic imaging can also be performed at the same time since a PW laser is used for the ODV.

## 1. Introduction

Sonoporation is depicted as the contrast agent-assisted ultrasound-induced transient permeability of the cell membrane [1]. It is often applied for enhancing the drug delivery efficacy during tumor therapy through the acoustic cavitation effect [2]. To overcome the limitation of instability during circulation in the bloodstream and to improve extravasation into the tumor through leaky tumor blood vessels (pore size 200–1200 nm) [3], nanodroplets and nanoemulsions were developed to replace microbubbles because of their stability and size advantages [4,5]. Perfluoropentane (PFP) is often used as the core of nanodroplets due to its low boiling point and biocompatibility. To trigger the cavitation effect from the nanodroplets, the method of inducing the droplet-to-bubble phase transition (i.e., vaporization) before inducing cavitation becomes an important issue.

It is known that droplets can be vaporized through acoustic droplet vaporization (ADV) and optical droplet vaporization (ODV). ADV exploits the acoustic pressure wave to initiate the vaporization process and has potential clinical applications for contrast-dependent imaging, emboli removal, drug delivery, and thermal ablation [6,7]. Specifically, when the sum of local pressure and Laplace pressure is below the vapor pressure of PFP at the rarefaction phase of the acoustic wave, it generates sufficient nucleation of the gas phase [8]. Note that although the boiling point of PFP is 29 °C at atmospheric pressure, the temperature for inducing phase transition of droplets is higher than 29 °C due to the presence of the Laplace pressure. Smaller droplets correspond to higher Laplace pressure and thus they need higher driven acoustic energy to produce bubble-forming nuclei [9,10]. Additionally, the longer pulse duration is also possible to activate the vaporization through heat absorption by the droplets [11]. The driving frequency is also reported as a factor to determine the pressure threshold of ADV, and the droplet size is related to the vaporization threshold but not the inertial cavitation threshold [9,12,13,14,15,16].

ODV is an alternative way to activate the liquid-to-gas phase transition of droplets encapsulating optical absorbing material by using laser irradiation instead of ultrasound exposure [17,18,19,20]. When the absorbed optical energy is above a specific threshold, it leads to a local temperature increase required for the vaporization. ODV-based applications also include imaging and therapy [21,22]. Moreover, the applications can be extended to dual mode photoacoustic and ultrasound imaging and image-guided cancer therapy [19,23,24,25]. In addition, both ADV and ODV can be employed for vaporization-directed sonoporation to deliver therapeutic agents into the cells. We previously combined near-infrared continuous wave (CW) laser with focused ultrasound to induce ADV and ODV of gold nanorod-encapsulated nanodroplets (AuNDs) for gold nanorod delivery, inertial cavitation, and the subsequent photothermal therapy [26]. Nonetheless, a relatively high powered CW laser (2 W/cm^2^ at 808 nm) was used and thus raises safety concerns during therapy. 

Instead of a combination of CW laser with ultrasound, it was recently reported that through synchronizing the ultrasound and PW laser on gold nanoparticles, the required laser fluence or acoustic peak negative pressure (PNP) can be lower to reduce the vaporization threshold [27,28,29,30,31,32]. For example, by using perfluorohexane nanoemulsions (boiling point 50 °C) with gold nanospheres encapsulated as the contrast agent, it was reported that when simultaneously stimulated with ultrasound and pulsed wave (PW) laser, the driving PNP for inducing cavitation nuclei can be reduced from 1.5 MPa (MI = 1.35) to 0.97 MPa (MI = 0.87) [30,31,32]. The study also demonstrated that the cavitation threshold can be effectively decreased when the laser pulse was at the peak negative pressure phase of ultrasound (i.e., rarefaction) than at the peak positive pressure phase (i.e., compression). This approach was successfully applied for improving photoacoustic imaging sensitivity and disruption of a blood clot in an in vitro study, but a large laser fluence of 87 mJ/cm^2^ was required [32]. In the present study, we test the feasibility of this approach for sonoporation. In order to better understand the underlying mechanisms and to optimize the sonoporation effects in vitro, we investigate vaporization and cavitation events using the proposed approach with various parameters including laser fluence, laser PRF, and acoustic pressure. In addition, the relationship between the sonoporation rate and vaporization/inertial cavitation dose is also established. Moreover, we focus on studying parameters for repeatable sonoporation. It helps to explore the potential for drug and gene delivery as well as feasibility as a new theranostic approach. This technique also has the benefits for performing photoacoustic imaging at the same time with sonoporation induced by ADV and ODV, and thus it is potentially an effective image-guided therapy approach. 

## 2. Materials and Methods 

### 2.1. Synthesis of AuNDs

The main components of AuNDs were gold nanorods (AuNRs), with longitudinal plasmon resonance wavelength at 808 nm, PFP (C_5_F_12_), and 20% human serum albumin solution (Octapharma AG, Lachen, Switzerland). The AuNDs were synthesized as reported in our previous work [26]. In brief, the three components were mixed in 1 mL of phosphate-buffered saline (PBS, purchased from Gibco, Thermo Fisher Scientific, Waltham, MA, USA) followed by sonication using a digital sonifier (Branson, Danbury, CT, USA) with a cup-horn sonotrode (Branson, Danbury, CT, USA). After 4 cycles of 5-min sonication and 5-min rest on ice, the droplet emulsions were formed. The sedimentary droplet emulsions were then resuspended in 1 mL of PBS and then centrifuged at 1700 rpm for 3 min for three times at 4 °C to isolate the nano-sized droplets. After centrifugation, the supernatant was further analyzed by using Coulter MultiSizer III (Beckman-Coulter, Hamburg, Germany) and Zetasizer (Nano Z, Worcestershire, UK) to count the number of the droplets and measure the size distribution, respectively. AuNDs with a size ranged from 200 to 500 nm were then applied for the following experiments.

### 2.2. Phantom Design and Wide Focused Laser Beam Setup

**Figure 1:**
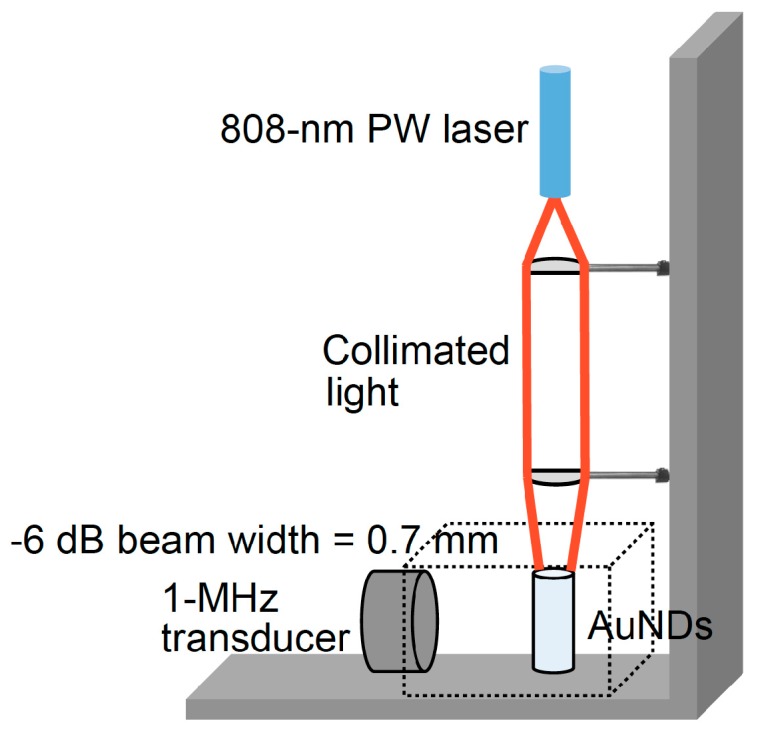
Laser beam setup.

To mimic the acoustic properties of biological tissues, we made an agar-based soft tissue phantom with 2% agarose gel. A columnar hole was created considering the confocal approach during the following experiments, which the distance between the hole and two sides of the phantom corresponding to the focal depth of the 1-MHz transmitting ultrasound transducer and the two receiving transducers. The diameter and height of the hole were 6 mm and 8 mm, respectively. The laser was irradiated from the top of the hole for the stimulation to the AuNDs. To cover the AuND-contained area, we established a wide-focused laser beam setup for delivering a 6-mm-wide laser beam (Figure 1). The diverged 808-nm laser beam was passed through a plano-convex lens to generate a collimated light and then directed to another plano-convex lens to generate a focused laser beam (−6 dB width = 0.7 mm). Note that the laser beam approximately has a Gaussian profile. In other words, the laser energy is the strongest at the beam center and becomes weaker as it moves away from the focal point. ODV only occurs near the center of the beam. Therefore, the effective area of the laser beam that can induce ODV is noticeably smaller than the −6 dB width. Thus, it is significantly smaller than the ultrasound wavelength at 1 MHz.

### 2.3. Experimental Setup

**Figure 2:**
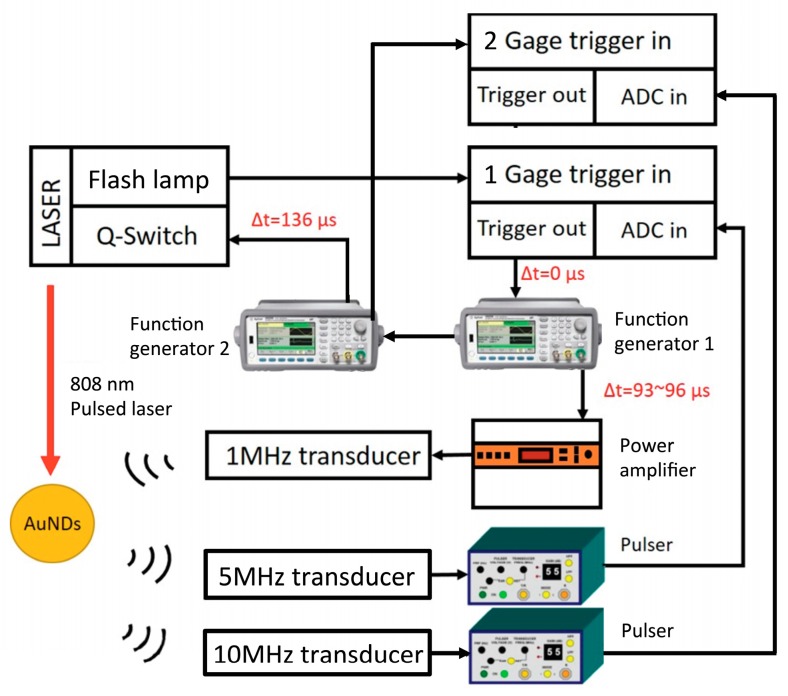
Experimental setup for triggering vaporization and inertial cavitation.

The experimental setup is illustrated in Figure 2. A 20-Hz TTL signal sent from the flash lamp of the laser was used as the main clock in the system. When the flash lamp signal starts to run, the signal is used to trigger the ADC board (CompuScope 14200, Gage, Lockport, NY, USA) that was controlled by LabVIEW. The ADC board then sends a trigger out immediately once an input trigger is received. Subsequently, the signal activates the function generator to send another trigger to the second function generator. Once an input trigger is received, this function generator then sends out a trigger delayed by 136 µs to trigger the laser Q-switch. The first function generator then generates a 1 MHz, 10-cycle sine wave that was delayed from 93 µs to 96 µs for investigating the signals collected from the 7^th^ to 10^th^ cycle of the ultrasound propagating waves and amplified using a power amplifier (250A250A, Amplifier Research, Souderton, PA, USA) to drive the transmitting focused ultrasound transducer (V302, Panametrics-NDT, Waltham, MA, USA, focused at 50 mm, f# = 1.96, 1 MHz). We used a 10 MHz ultrasound transducer (V327, Panametrics-NDT, Waltham, MA, USA) focused at 30 mm and a 5 MHz unfocused ultrasound transducer (V310, Panametrics-NDT, Waltham, MA, USA) to receive the cavitation signals and the 2^nd^ to 4^th^ harmonic signals, respectively. The signals were recorded by the ADC at a 100-MHz sampling rate and the data were saved on the computer for further analysis. An 808-nm laser beam was generated from a wavelength-tunable OPO laser (Opolette 532, OPOTEK, Carlsbad, CA, USA) to produce 10-ns duration laser pulses. The laser fluence was measured by a power meter (Nova II, Ophir, Jerusalem, Israel) at the focal site of the ultrasound transducer. In Figures 5,6 and 9, the relative laser delay time indicates the time allowing for the laser beam and ultrasound wave to arrive at the sample at the same time (e.g., 96 µs). For photoacoustic (PA) imaging, a linear ultrasound array transducer (L7.5-12840, 128 elements, S-Sharp, New Taipei City, Taiwan) interfaced with a programmable imaging system (Prodigy, S-Sharp, New Taipei City, Taiwan) was used for receiving PA signals and performing B-mode imaging. All signal and image processing was performed in Matlab (R2015b, The MathWorks, Natick, MA, USA).

### 2.4. Vaporization Signals Detection

**Figure 3:**
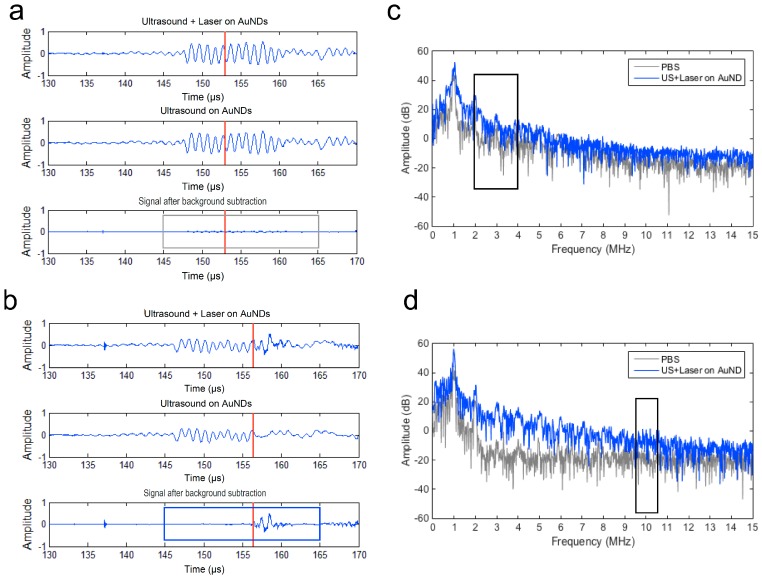
Scatter signal detection of vaporization and cavitation events using a 5 MHz unfocused transducer and a 10 MHz focused transducer. The red line denotes the lasing time. The background signal was detected using ultrasound only. (**a**) No vaporization and cavitation event was detected. (**b**) Vaporization and cavitation events were detected. (**c**) Spectra of the received vaporization signals shown in the gray box in (a) and the blue box in (b). (**d**) Spectra of the received cavitation signals shown in the gray box in (a) and the blue box in (b). Black boxes in (c) and (d) denote the range of the spectra for root-mean-square (RMS) amplitude calculations.

When the droplets have vaporized to gas bubbles, acoustic scattering is significantly stronger than that of the droplet and causes an increase in echogenicity. Thus, the growth of the bubbles during the vaporization causes an increase in power over time [33]. Upon vaporization, the vaporized droplets generate higher harmonics of the scattered signals due to the nonlinearity [13,34]. Accordingly, here we analyzed the power of the second to fourth harmonics in the frequency domain (n*f; *n* = 2, 3, 4; *f* = 1 MHz) to estimate the 2^nd^ to 4^th^ harmonic root-mean-square (RMS) values. Figure 3 shows that a total of 20 µs around the laser pulse (red line) was selected for data analysis (Figure 3a: without vaporization, Figure 3b: with vaporization). After the background signals of PBS were subtracted from the received signals, the remaining signals were used for calculating the 2^nd^ to 4^th^ harmonic RMS values in the frequency domain to determine the vaporization occurrence (Figure 3c). 

### 2.5. Differential Inertial Cavitation Dose and Acoustic Pressure Measurement 

The cavitation effect was quantified by calculating the differential inertial cavitation dose (dICD). A 10-MHz transducer was used to receive the broadband noise as the cavitation events. ICD was calculated as the RMS values of the spectrum between 9.5 MHz and 10.5 MHz from the received signals [35]. In each individual experiment, 200 firings were calculated. Briefly, it was calculated as the area under the time-amplitude curve over the entire recording time period, after the subtracting the background time-amplitude curve (i.e., baseline subtraction). The resulting amplitude was denoted as dICD values. A Gaussian time window was applied on the residual signals to obtain a time-frequency view of the spectrum before each ICD calculation. The acoustic field of the transmitting 1-MHz ultrasound transducer was calibrated using a needle-type hydrophone (MHA9-150, FORCE Technology, Denmark) for measurements of the peak negative pressure (PNP).

### 2.6. Sonoporation Rate and Cell Death Rate Measurement

In comparing the data to our previous study [26], we kept using BNL cells, a mouse hepatocarcinoma cancer cell line, as an in vitro cell model for sonoporation analysis. It was cultured with DMEM containing 10% fetal bovine serum plus 0.1% penicillin/streptomycin (all purchased from Gibco, Thermo Fisher Scientific, Waltham, MA, USA) at 37 °C, 5% CO_2_ incubator. To monitor the sonoporated cells in situ, a membrane impermeant fluorescence dye, propidium iodide (PI) (eBioscience, Thermo Fisher Scientific, Waltham, MA, USA) with max excitation/max emission wavelength at 535/617 nm was used for investigating the fluorescent dye influx into the cells when the cell membrane is transiently disrupted under sonoporation [36,37]. It is a ready-to-use solution and was added into cellular suspensions with a 1:50 dilution first before processing sonoporation. Then, 100 µl of cells/PI mixture was mixed well with 100 µL of AuNDs. All mixtures were prepared at the same time and then divided into aliquots of 200 µl for the subsequent experiments. The final concentration of cells was 2 × 10^6^ cells/mL and was 2 × 10^8^ droplets/mL for AuNDs. The experimental setup was the same as that in the cavitation measurements. For the negative control (NC) group (i.e., cells without any treatment), we took one of the aliquots and also loaded it into the hole of the phantom but with no treatment of laser and ultrasound. After exposure of laser and ultrasound, samples were collected from the holes of the phantom. After each batch of the experiments was done, we washed the hole with PBS at least three times. To ensure the hole was fully cleaned, we checked the background signals of the PBS before the next experiments. Only when the background signals were similar to that in the initial experiments (i.e., unused hole), the experiments and data collection can continue. For the experiments studying the sonoporation as a function of the relative laser delay time, samples were collected every 0.2 µs of the laser delay time after the treatment. To differentiate between dead cells and sonoporated cells from the treated cells, after the treatment, cells were further counterstained with Calcein-AM viability dye (Molecular Probes, Invitrogen, Carlsbad, CA, USA) with max excitation/max emission wavelength at 495/515 nm for 10 min on ice. As a cellular index, Hoechst 33342 membrane-permeable nucleic acid dye (NucBlue, Molecular Probes, Invitrogen, Carlsbad, CA, USA) with max excitation/max emission wavelength at 360/460 nm was also added into the suspensions together with Calcein-AM. Ice-cold PBS was then used for washing out the excess dye in the mixture. The sonoporation rate was investigated under an inverted microscope (IVM-2A, SAGE Vision, New Taipei City, Taiwan). The sonoporation rate was determined as the proportion of cells that emit positive PI red fluorescence/positive Calcein-AM green fluorescence to the cells that emit Hoechst 33342 blue fluorescence. The cell death rate was determined as the proportion of the cells that emit positive PI red fluorescence/negative Calcein-AM green fluorescence to the cells that emit Hoechst 33342 blue fluorescence.

## 3. Results

### 3.1. Characterization of AuNDs

**Figure 4:**
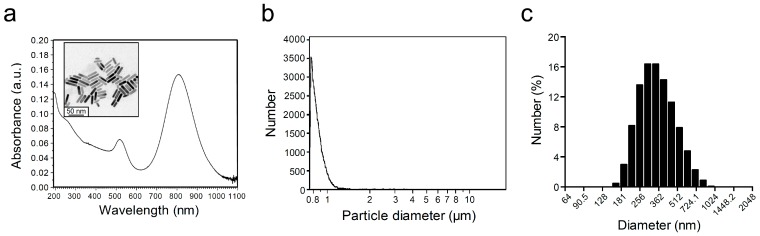
The size distribution of gold nanodroplets. (**a**) The spectrum map shows that the gold nanords (AuNRs) used for gold nanodroplet (AuND) synthesis has a peak optical absorbance wavelength at 808 nm. (**b**,**c**) The size distribution of AuNDs measured by using Multisizer and Zetasizer, respectively.

According to the optical spectrum, AuNRs with an aspect ratio at four were used for encapsulation into nanodroplets with a longitudinal surface plasmon resonance wavelength of 808 nm (Figure 4a). After centrifugation, the size of the AuNDs measured by the Multisizer was generally less than 1 µm, and according to the data collected from Zetasizer, the size distribution of AuNDs mainly ranged from 200 to 500 nm in diameter (Figure 4b,c). 

### 3.2. Synchronized Optical and Acoustic Droplet Vaporization

**Figure 5:**
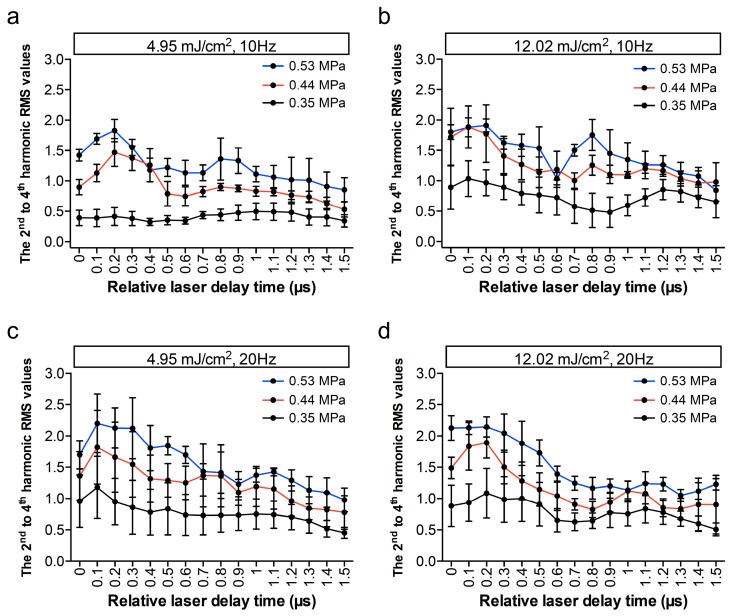
AuNDs vaporization triggered using ultrasound and laser simultaneously. The 2^nd^ to 4^th^ harmonic RMS values were plotted as a function of relative laser delay time. In each individual experiment, 200 recordings were collected at (**a**) 4.95 mJ/cm^2^, 10 Hz; (**b**) 12.02 mJ/cm^2^, 10 Hz; (**c**) 4.95 mJ/cm^2^, 20 Hz; and (**d**) 12.02 mJ/cm^2^, 10 Hz. Lines labeled in different colors denote different driving acoustic PNPs in each figure. Each point indicates the mean and SD for three individual experiments.

To find the optimal condition for triggering the vaporization of AuNDs, we tested various parameters including different laser fluence and different laser PRF for inducing ODV. Several studies have reported that the sufficient PNP is a critical factor for induction of the ADV [9,11,33], and thus we also applied three different driving acoustic PNP for each batch of AuND samples for inducing ADV. In addition, these parameters were selected according to our previous study in order to avoid unwanted cell death [26]. As shown in Figure 5, under the same driving PNP and comparing the laser fluence of 12.02 mJ/cm^2^ to 4.95 mJ/cm^2^, the 2^nd^ to 4^th^ harmonic RMS values were at a similar level when laser PRF was 20 Hz, and the values were higher when the PRF decreased to 10 Hz. Under the same driving PNP and comparing the laser PRF of 20 Hz to 10 Hz, a similar vaporization level was found when the laser fluence was 12.02 mJ/cm^2^, and it was higher when the laser fluence decreased to 4.95 mJ/cm^2^. For triggering ADV, under the same laser fluence and PRF, a higher PNP can trigger more vaporization events. Moreover, when we plotted the vaporization events from the calculated values at each of the relative laser delay time points, it displayed a 1-µs period, which corresponded to the period of the 1-MHz acoustic wave. These results indicate that laser fluence, PRF, and PNP can mediate the ODV or ADV events of AuNDs. Notably, the 1-µs periodical dynamic suggests that the synchronization of ODV and ADV using this developed method can potentially trigger the vaporization at the ultrasound rarefaction phase at lower vaporization thresholds.

### 3.3. Periodical Cavitation Dynamics and the Relationship between Vaporization and Cavitation

**Figure 6:**
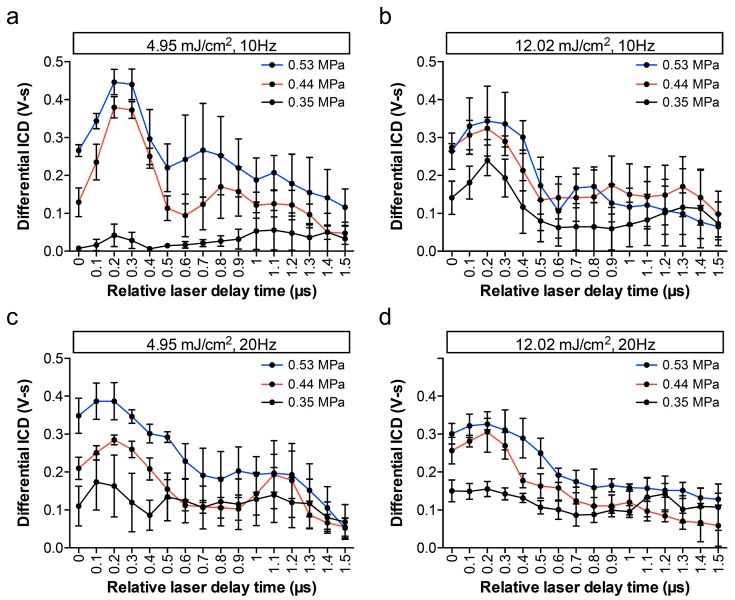
AuNDs cavitation triggered using ultrasound and laser simultaneously. The differential inertial cavitation dose (ICD) values were plotted as a function of relative laser delay time. In each individual experiment, 200 recordings were collected at (**a**) 4.95 mJ/cm^2^, 10 Hz, (**b**) 12.02 mJ/cm^2^, 10 Hz, (**c**) 4.95 mJ/cm^2^, 20 Hz and (**d**) 12.02 mJ/cm^2^, 10 Hz. Lines labeled in different colors denote different driving acoustic PNPs in each figure. Each point indicates the mean and SD for three individual experiments.

**Figure 7:**
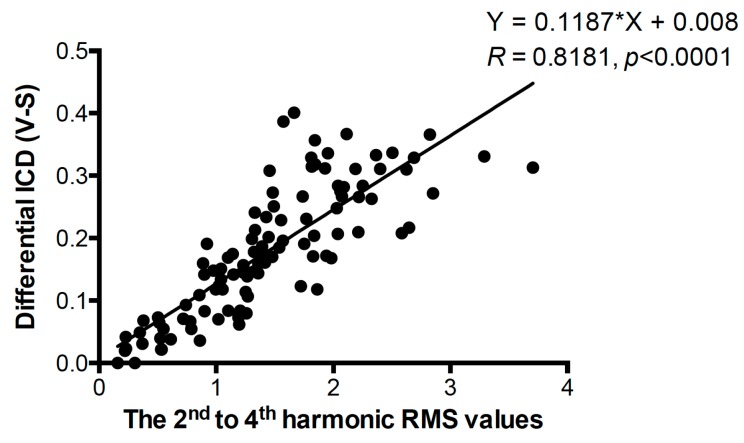
Correlation of differential ICD values versus the 2^nd^ to 4^th^ harmonic RMS values. The correlation coefficient (*R*), the *p*-value of the Pearson’s correlation test, and the equation of the linear regression line are shown around the line. *n* = 105.

The same signals received by the same setup for detecting vaporization were utilized for further analyzing the inertial cavitation events as shown in Figure 6. When the laser fluence was only 4.95 mJ/cm^2^ and the PRF was 10 Hz, the dICD was barely detected (Figure 6b). We further examined whether the driven PNP affect the synchronized vaporization-assisted inertial cavitation or not. Comparing PNP of 0.53 MPa, 0.44 MPa, or 0.35 MPa, the dICD values were one- to three-fold higher in each laser parameter set. More importantly, except at the low laser fluence and PRF group (i.e., 4.95 mJ/cm^2^, 10 Hz), dICD values in other groups were changed with a 1-µs period, which agreed with the analysis of the vaporization events. To correlate the relationship between vaporization and inertial cavitation events, Pearson’s correlation test and a fitted linear regression method were performed (Figure 7). All data collected from the AuNDs exposed under laser PRF of 20 Hz, laser fluence of 12.02 mJ/cm^2^, and PNP of 0.53 MPa were selected for the calculation. The correlation coefficient (*R*) of dICD values versus the 2^nd^ to 4^th^ harmonic RMS values was 0.8181 (*p* < 0.0001). The significantly high correlation reveals a high positive correlation between vaporization and inertial cavitation.

### 3.4. Comparison of Vaporization and Cavitation Events

**Figure 8:**
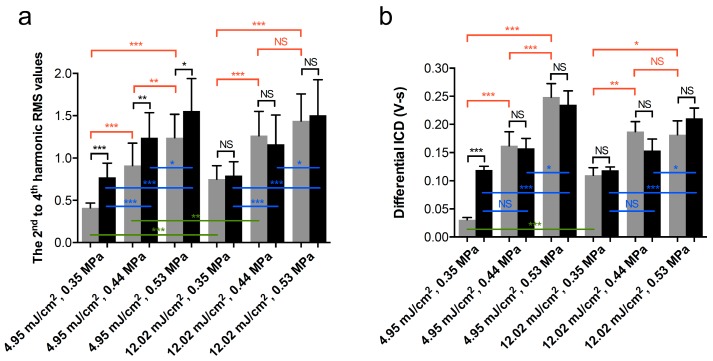
Comparison of vaporization and cavitation events under different parameter sets. (**a**) The 2^nd^ to 4^th^ harmonic RMS values and (**b**) differential ICD values as a function of different exposure parameters. Gray columns, laser PRF = 10 Hz. Black columns, laser PRF = 20 Hz. Each column indicates the mean and SD from three individual experiments, *n* = 48. Student’s *t*-test was applied for the determination of the significant difference between two sets of data. *, *p* < 0.05; **, *p* < 0.01; ***, *p* < 0.001; NS, no significance. Green lines denote the comparison of the two data sets between low and high laser fluence, only the three labeled groups display significant differences. Black lines denote the comparison of the two data sets between low and high laser PRF. Red lines and blue lines denote the comparison of the two data sets between different acoustic PNPs in the groups of laser PRF of 10 Hz and 20 Hz, respectively.

To further evaluate the respective contribution of laser fluence, laser PRF, and acoustic PNP to vaporization and cavitation, the 2^nd^ to 4^th^ harmonic RMS values or the dICD values of each data set at each relative laser delay time point were averaged in a single group. As shown in Figure 8a, under the laser PRF of 20 Hz, no difference in vaporization events between laser fluence of 4.95 mJ/cm^2^ and 12.02 mJ/cm^2^ was observed. In the groups of laser PRF at 10Hz, higher laser fluence significantly enhanced the vaporization values under PNP of 0.35 MPa and 0.44 MPa, but no enhancement was found when the driving PNP was increased to 0.53 MPa (Figure 8a, green lines). Under a laser fluence of 4.95 mJ/cm^2^ and comparing a laser PRF of 20 Hz to 10 Hz, higher laser PRF significantly enhanced the vaporization signals in each parameter set. However, no significant difference was found in the groups with a laser fluence of 4.95 mJ/cm^2^ (Figure 8a, black lines). No matter what the laser fluence or PRF was, almost all data sets showed that a higher PNP can significantly enhance the vaporization signals, suggesting that PNP is also a crucial factor to induce ADV (Figure 8a, red and blue lines).

In Figure 8b, in comparison with the contribution of the laser fluence and PRF to AuNDs cavitation between different data sets, the higher laser fluence only significantly enhanced the cavitation events under a PNP of 0.35 MPa and a PRF of 10 Hz (Figure 8b, green lines), and a higher laser PRF significantly enhanced the cavitation events under a PNP of 0.35 MPa and a laser fluence of 4.95 mJ/cm^2^ (Figure 8b, black lines). When studying the contribution of PNP for inducing AuND cavitation, most of the data sets showed that the higher PNP can significantly enhance the cavitation signals, indicating that the PNP is important to induce AuND cavitation (Figure 8b, red and blue lines). Moreover, it was found that 0.44 MPa was the threshold to induce cavitation when the laser fluence was 4.95 mJ/cm^2^.

### 3.5. Synchronized ODV- and ADV-Based Sonoporation

**Figure 9:**
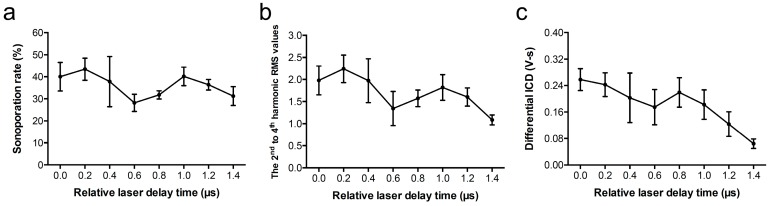
Sonoporation of BNL cells triggered using ultrasound and laser simultaneously. (**a**) Sonoporation rate, (**b**) the 2^nd^ to 4^th^ harmonic RMS values, and (**c**) differential ICD values as a function of relative laser delay time. In each individual experiment, 200 recordings were collected for the analysis. Each point indicates the mean and SD from three individual experiments.

We further explored if the synchronized vaporization-assisted inertial cavitation can enhance the sonoporation. In this experiment, the laser fluence was fixed at 12.02 mJ/cm^2^, the laser PRF was at 20 Hz, and the PNP was at 0.53 MPa. The results again showed that the change of the sonoporation rate was in agreement with the 1-µs ultrasound period (Figure 9). 

**Figure 10:**
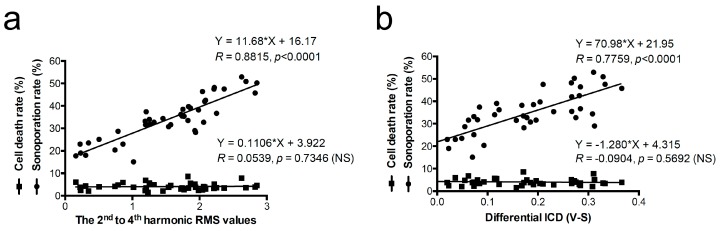
Correlation of the cell death rate and the sonoporation rate versus differential ICD values and the 2^nd^ to 4^th^ harmonic RMS values. The correlation coefficient (*R*), the *p*-value of the Pearson’s correlation test, and the equation of the linear regression lines were shown around the lines. *n* = 42 in each line. NS: no significance.

According to the results of the Pearson’s correlation test and a fitted linear regression method, a significantly high correlation between sonoporation and the 2^nd^ to 4^th^ harmonic RMS values or dICD values was demonstrated (Figure 10, *R* = 0.8815 and 0.7759, respectively; *p* < 0.0001). Concerning cell death that occurs after US and laser treatment and that may affect calculations of the sonoporation rate, we used PI+/Calcein- to rule out the dead cells. Results showed that the correlation between the cell death rate and the 2^nd^ to 4^th^ harmonic RMS values or dICD values was very low for both, and no significant correlation was found (Figure 10, *R* < 0.1, *p* > 0.5). It indicates that both vaporization and cavitation can trigger sonoporation, and the parameters we used were not able to induce significant cell death. The sonoporation occurred when the vaporization or cavitation level was high, which may also correspond to the rarefaction phase of the transmitted ultrasound wave.

**Figure 11:**
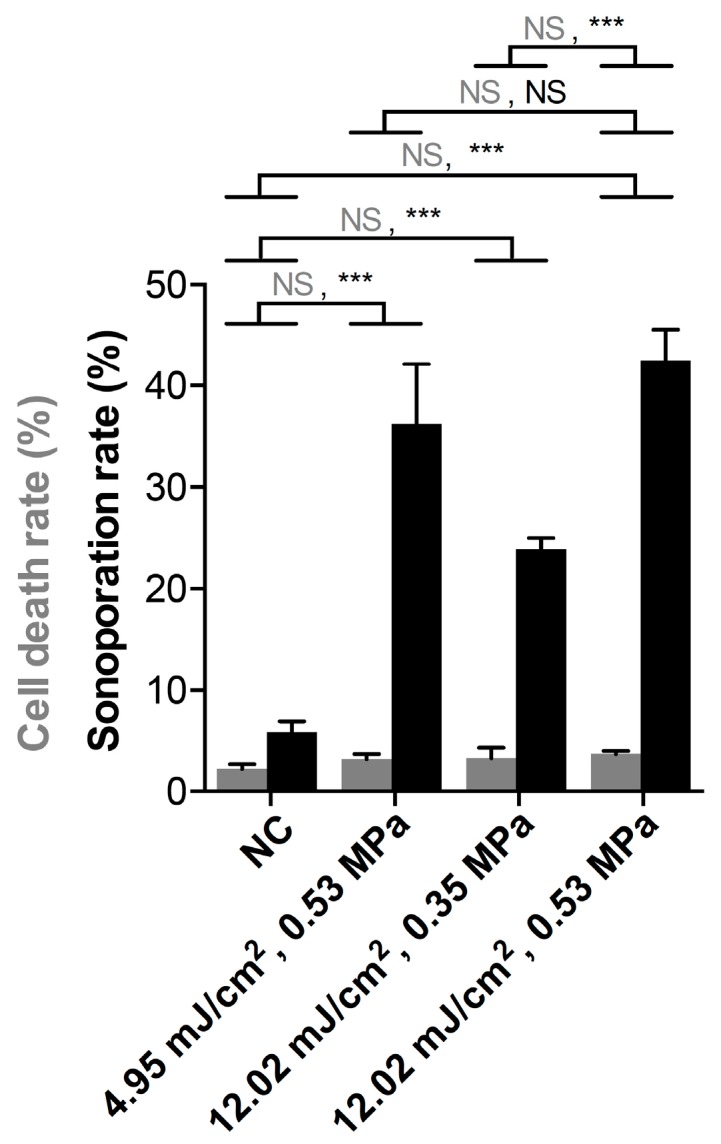
Comparison of sonoporation rate and cell death under different parameter sets. The laser PRF was fixed at 20 Hz in the experiments. Gray columns: cell death rate. Black columns: sonoporation rate. NC: negative control group. Student’s *t*-test was applied for determining the significant difference between the two sets of data. ***, *p* < 0.001; NS: no significance. At least 350 cells were counted in each individual experiment. Each column indicates the mean and SD from three individual experiments.

Here, we further discuss the role of ODV and ADV in sonoporation and cell death. In comparison with the negative control group (i.e., no exposure to acoustic and optical energy), cells treated with laser fluence or PNP can significantly induce sonoporation but not cell death. Under the laser fluence of 12.02 mJ/cm^2^, the sonoporation rate was significantly enhanced due to the higher PNP. Under a PNP of 0.53 MPa, no difference in sonoporation rate between a laser fluence of 4.95 and 12.02 mJ/cm^2^ was found (Figure 11). The results indicate that in addition to the laser fluence, the driving PNP, which was the main factor to enhance both ADV and cavitation, was also a critical factor to enhance sonoporation. 

### 3.6. Photoacoustic Imaging

**Figure 12:**
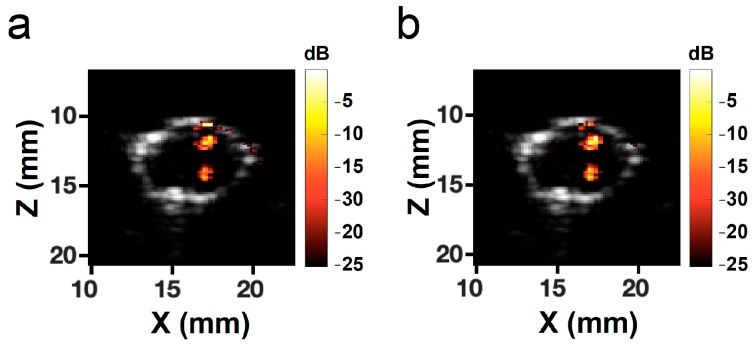
Photoacoustic imaging of (**a**) AuNRs and (**b**) AuNDs. The grayscale images in both panels denote the B-mode images of the cross-section of the hole in the phantom. Color bars represent the intensity of the photoacoustic signals. Locations of the AuNRs and the AuNDs are clearly visible.

We further used AuNDs as the photoacoustic contrast agent to test the feasibility of performing PA imaging under the same setup. Under a laser fluence of 12.02 mJ/cm^2^, PA signals were successfully received by using a 7.5-MHz linear array transducer (Figure 12). The PA signal intensities obtained from AuNRs and AuNDs were at the same level, indicating that the ability to perform PA imaging of AuNDs using the same setup was possible.

## 4. Discussion

Nanodroplets are generally stable because of the small size and the shell surface tension. Relatively large acoustic energy and/or high laser energy are needed to trigger vaporization. The aim of the present study was to provide a more efficient vaporization approach by synchronizing ODV with ADV for reducing the cavitation threshold and improving the therapeutic effects with fewer safety concerns and higher sonoporation efficiency. It has been reported that delivering laser pulses at the negative ultrasound peaks can reduce the vaporization and cavitation threshold of gold nanoemulsions [30,32]. In this study, we further demonstrated improved sonoporation effects and successfully demonstrated a synchronization scheme for cavitation. The relatively large laser beam width shows that strong optical focusing with the optical resolution is not needed, thus it can potentially be used for in vivo applications.

In previous studies, for ODV-assisted in vitro fibrin clot disruption, 500 mJ/cm^2^ of laser fluence was required [32]; for ODV-assisted in vitro cancer cell destruction, 90 mJ/cm^2^ of laser fluence was required [19]. Based on our results, when ODV is synchronized with ADV, the laser fluence required for triggering ODV using the wide laser beam setup was decreased to 4.95 mJ/cm^2^. For the FDA-approved laser use in class IIIB (i.e., medium-power invisible laser), the laser energy should be less than 125 mJ and the pulse duration should be less than 0.25 s. In the current setup, the pulse duration of the OPO pulsed laser was only 10 ns, even under the condition of laser fluence at 12.02 mJ/cm^2^, the energy per pulse was only 3.4 mJ. Our studies showed that the lowest PNP for triggering the ADV and inertial cavitation is 0.35 MPa (MI = 0.35), which can be readily implemented in the in vivo therapeutic applications under FDA safety limits (maximum MI must be 1.9 or less) for diagnostic ultrasound. Accordingly, both the laser and ultrasound setup did not have any safety concerns. The low cell death rate shown in Figure 10 also demonstrated safety when using this technique. As reported in the literature, synchronization of the laser excitation and ultrasound pulse can extend the lifetime of the cavitation bubbles [32], which may further benefit the nanodroplet-based photoacoustic imaging and therapeutic applications. Furthermore, since photoacoustic imaging can be performed at the same time with the PW laser used for ODV, image-guided therapy is possible with our approach.

It was reported that the threshold of inertial cavitation and ADV is comparable [11]. When introducing exogenous cavitation nuclei, it was also reported that the threshold of ADV can be effectively decreased [38]. In Figure 7, the significantly high positive correlation between vaporization and inertial cavitation indicates that the induction rate of these two AuND behaviors is in good agreement with each other. When we synchronized PW laser with ultrasound stimulation, not only can the ODV and ADV be synchronized for reducing the vaporization threshold, the acoustic pressure can further introduce more cavitation nuclei to trigger vaporization. 

When AuNDs were exposed under laser and ultrasound energies without incubation with cells (Figure 5), the values calculated from vaporization events gradually decreased over the relative laser delay time. It was due to the fact that vaporized AuNDs were destructed by inertial cavitation, thus causing a reduction of the amount of AuNDs over time. The reduction was not obvious in Figure 9 due to the interaction between the cells and the AuNDs which somewhat stabilized the vaporized AuNDs. At the rarefaction phase (i.e., relative laser delay of 0.2 µs), the vaporization values in both conditions were around 2.1 but the dICD value was 0.32 in the AuNDs only group (Figure 5) and was reduced to 0.24 in the sonoporation group (Figure 9), indicating 25% reduction of the cavitation events. We believe that was the reason for the difference between the two groups regarding periodicity.

In Figure 8 and Figure 11, vaporization, cavitation, and sonoporation level were all enhanced when increasing the PNP. In Figure 8, the higher laser PRF enhanced vaporization signals rather than the cavitation signals, especially in the lower laser fluence group. The AuNDs encapsulated plasmonic gold nanoparticles, which can absorb electromagnetic energy from the laser to generate localized heating required for ODV [21,26]. When the time interval between two laser pulses is less than the time required for diffusion of heat (i.e., when PRF is high), the heat accumulates inside the focal volume [39,40]. Thus, in our data, we found that when the laser fluence was low, a higher PRF was an important factor for triggering vaporization under different driving PNPs. When PNP was over 0.44 MPa, even the vaporization events had a significant increase comparing high PRF to low PRF, and such enhancement cannot significantly induce more cavitation events. However, when the PNP was only 0.35 MPa, the laser fluence only 4.95 mJ/cm2, and the laser PRF only 10 Hz, both vaporization and cavitation events were too low, which caused a two-fold and four-fold increment in comparison with the high PRF groups. It suggests that the accumulated heat generated by high PRF was critical for both vaporization and cavitation under such experimental conditions. However, when the laser PRF was lower, vaporization and cavitation were barely induced if the laser fluence and PNP were also lower. The acoustic PNP was an essential factor to mediate ADV, and laser fluence and laser PRF were critical to mediate ODV. Thus, our study demonstrated that ADV synchronized with ODV was beneficial to facilitate nanodroplet vaporization-, cavitation-, and sonoporation-based applications. Sonoporation refers to the transient pore formation of the cell membrane through the interaction between the contrast agent and the cell membrane [1]. If the membrane resealing time is over 20 min or the pore size was too large, the cells will die because of the irreversible harm to the cells [41,42]. In Figure 11, no significant cell death was found, indicating that the sonoporation-directed pore formation was transient and reparable with the proposed technique and the associated parameters.

Our results indicate that we can use this method to decrease the energy required for sonoporation-directed applications. For example, lower energy can avoid unwanted cell damage for sonoporation-based gene therapy or cancer therapy. Figure 10 shows that when treating AuNDs with this synchronization method, the sonoporation rate in most of the groups was significantly enhanced in comparison to the non-treatment group. It suggests that synchronizing ODV and ADV can effectively enhance the sonoporation-directed membrane pore formation, and the cavitation effect triggered by the reduced threshold is sufficient to induce sonoporation. In other words, we can effectively induce the sonoporation with a reduced threshold. Furthermore, the high correlation coefficient indicates that higher cavitation effect can induce more sonoporation events (Figure 11). This conclusion is also consistent with the previous study [35]. In other words, it suggests that when we triggered higher cavitation effect at the ultrasound rarefaction phase, more sonoporation events can be induced. Future work will focus on the optimization of a stable and controllable method to induce sonoporation and thus improve the delivery efficiency of therapeutic agents. Furthermore, the associated inertial cavitation effect can be applied for multi-mode therapy at the same time and position, such as cancer cell destruction or tumor vasculature disruption.

Note that the actual energy delivered into the region of interest for in vivo applications can be very different from those for in vitro applications. This is mainly due to the scattering and attenuation of biological tissues. Moreover, to enhance the targeting specificity in tumor therapy, AuNDs conjugated with antibodies that can recognize the tumor-enriched proteins will be beneficial. Specifically, the synchronized vaporization-assisted sonoporation can potentially enhance drug delivery, and the associated inertial cavitation can further destruct the tumor tissue at the same time. Furthermore, photoacoustic imaging can be performed for monitoring the sonoporation process at the same time as induced ODV and ADV. Such synergetic multi-mode guided therapy can be useful in future clinical applications.

## 5. Conclusions

Sonoporation has been demonstrated as an effective way for drug or gene delivery. This study used AuNDs as the contrast agent and synchronized the laser excitation and ultrasound pressure wave to synchronize the ODV and ADV of AuNDs. We successfully used this method to improve the vaporization-assisted cavitation and sonoporation in vitro. We found that delivering laser energy at the rarefaction phase of the ultrasound pressure wave was about two-fold effective. The driving PNP of ultrasound was not only critical for modulating inertial cavitation but also dominated the sonoporation efficacy. The reduced acoustic pressure and optical energies make this a potential method for in vivo applications for imaging-guided drugs or gene delivery. Based on our study, a multi-mode theranostic method can also potentially be performed.
